# Genome-wide association studies reveal the genetic basis of growth and carcass traits in Sichuan Shelduck

**DOI:** 10.1016/j.psj.2024.104211

**Published:** 2024-08-14

**Authors:** Zhao Yang, Yang Xi, Jingjing Qi, Liang Li, Lili Bai, Jun Zhang, Jia Lv, Bo Li, Hehe Liu

**Affiliations:** ⁎ Farm Animal Genetic Resources Exploration and Innovation Key Laboratory of Sichuan Province, College of Animal Science and Technology, Sichuan Agricultural University, Chengdu 610000, China; † Rural Revitalization Development Service Centre, Zigong, China; ‡ Farming Service Centre, Rong County, Zigong, China

**Keywords:** GWAS, Sichuan Shelduck, growth, meat production performance, candidate gene

## Abstract

China has abundant local duck resource populations, and evaluating the characteristics of these breeds will help improve development and utilization. In this study, we conducted the first investigations of growth and slaughter performance on Sichuan Shelduck (n = 240), an endangered duck local breed. The average body weight is 1497.91 g at 90 d of age. According to the growth curve through data recorded every 2 wk, we observed a low relative growth rate (**RGR**) for the early growth stage. The RGR shows a decreasing trend with age increasing in the stage from 0 to 56 d of age. The SNP-based heritability estimation showed the growth rate has a relatively high heritability, indicating high genetic stability for this trait. In the correlation analysis, the percentage of leg muscle is positively correlated with the absolute growth rate (**AGR**) at 28 to 42 d of age, whereas it is negatively correlated with the earlier stages, exhibiting a time-specific correlation result. Additionally, genome-wide association studies (**GWAS**) identified *PCSK6, TOX2*, and *TOMM*7 as potential candidate genes influencing AGR (42–56) and AGR (56–90), while the candidate genes of slaughter traits were *PTP4A2, FAM110B, TOX, UBXN2B*, and *FCHSD2*. These results provide an important reference for further understanding the genetic basis of growth and meat production performance of Sichuan Shelduck.

## INTRODUCTION

Globally, particularly in Asia, ducks have become an important part of meat consumption and developed towards multi-type consumption ways (Tai and Tai, 2001; Magdelaine, et al., 2008; Whitton, et al., 2021). Consequently, breeders aim to develop diverse duck breeds to meet future market demands. The availability of abundant duck genetic resources is crucial for breeding improved varieties (Liu, et al., 2021). Investigation and screening of the genes underlying these excellent traits of local ducks can also provide a scientific basis for genetic improvement.

Recently, High-throughput sequencing and genome-wide association studies **(GWAS)** have been widely applied to farm animals to screen candidate genes and mutations related to duck growth and production traits (Ding et al., 2022; Li, et al., 2022). Notably, functional genes and loci have been discovered in various local duck breeds (Li, et al., 2023c). In terms of growth and development, whole-genome data elucidated the genetic pattern of Zhongshan ducks, highlighting the roles of *SEMA5B* and *MIB1* in growth and development (Chen, et al., 2023). In Jinding ducks, Shanma ducks, and Shaoxing ducks, Whole genome resequencing was used to identify growth-related genes (***IGF1R***) and skeletal development genes (***CDF5***) (Li, et al., 2023b). The researchers identified *EIF2AK3* gene was associated with body weight development in Shanma duck, Jinding duck, Gaoyou duck, Maple Leaf duck, Shaoxing duck (Zhu, et al., 2021). Studies on Gaoyou and Jinding ducks revealed that different expression patterns of the *MYOD* and *MYF6* genes in breast muscle and leg muscle may be related to muscle development and differentiation (Li, et al., 2014). In addition, Pekin duck is renowned globally as a high-quality meat breed and has been extensively studied (Zhang, et al., 2018). The GWAS results indicated that *IGF2BP1* gene was a key candidate gene affecting body size and feed efficiency in Peking ducks (Zhou, et al., 2018). And different genotypes of *MAGI3* may influence breast muscle development of Peking ducks, and then affect the thickness of the perimysium (Tang, et al., 2023). Zhu *et al*. showed that solute carrier proteins (*SLC39A10, SLC10A2*) may affect the growth of meat-type Peking ducks (Zhu, et al., 2019). Deng *et al*. identified *PLXDC2* associated with breast width and *TNS3* associated with fossil bone length (Deng, et al., 2019). In terms of slaughtering performance, *LOC101791418, TUBGCP3* and *ATP11A* play an important role in eviscerated weight and leg muscle weight percentage (Deng, et al., 2019). *CTDSPL* and *PKP1* are new candidate genes for breast muscle thickness used by genotyping-by-sequencing (Deng, et al., 2020). Furthermore, genotyping and GWAS analyses have identified 2 SNPs (chr29:2,296,787 and chr29:2,296,832) in the *AUTS2* gene that were related to high leanness in Pekin ducks (Liang, et al., 2023).

Despite the increasing demand for higher growth rates and meat production of ducks, the development and utilization of local duck resources have been neglected. The Sichuan Shelduck, well-known for its small body size, early maturity, strong adaptability, well-developed chest and leg muscles, and delicious meat, is a dual-purpose duck breed for meat and egg production. Combining its advantages with fast growth and high egg production from duck genetic resources can result in economically favorable hybridization combinations. However, there is currently a lack of comprehensive evaluation of growth and meat production traits, unclear genetic basis, and insufficient molecular markers, which limits its application in hybrid breeding. This study conducted a systematic evaluation of the growth performance and slaughter performance of both males and females. Additionally, we used GWAS to identify candidate genes and associated SNPs. The research results can enhance our understanding of the genetic basis of Sichuan Shelduck and promote its application in genetic improvement.

## MATERIALS AND METHODS

### Animals

All ducks utilized in this experiment were sourced from the Rong County Hemp Duck Breeding Farm in Zigong City, Sichuan Province. A total of 240 ducks were included for the assessment of growth performance, body size, and slaughter performance. These ducks were raised according to standard feeding procedures and nutritional requirements (Supplementary Table S1). The experimental ducks were initially kept in the brooding room for 0 to 14 d. From 15 to 90 d of age, the ducks were transferred to a ground flat breeding house for feeding. Throughout the experiment, the same feeding density and environment were maintained, and the ducks had access to food *ad libitum*. Body weight was measured every 2 wk from 0 to 90 d of age. At the age of 90 d, 240 ducks were randomly selected for slaughter, during which slaughter performance metrics, breast muscle samples, and fresh blood samples were collected for subsequent analyses. The Animal Care and Use Committee of the Institute of Animal Sciences, Sichuan Agricultural University, approved all animal experimentation and ducks. The guidelines approved the methods and protocols used in this study. The guidelines for the methods and protocols used in this study were also approved. All efforts were made to minimize any potential suffering experienced by the ducks.

### Collection of Phenotypic Value

*Size and weight.* Body weight gain between 2 weighing times is defined as the absolute growth rate **(AGR)**. The relative growth rate **(RGR)** is determined by the ratio of AGR to the previous weight. The calculation formulas are as follows (W_0_: measured weight at last time; W_1_: measured weight at next time):

$$AGR\;\left(g\right)=W_{1}-W_{0}$$

$$RGR\;\left(\%\right)=\frac{W_{1}-W_{0}}{W_{0}}\times 100\%$$

at 90 d of age, body measurements were collected for all ducks. Half-Divina dept **(H-Dd)**: Measured with a tape measure from the tip of the beak to the midpoint of the line connecting the hip bones. Neck length **(NL)**: Distance from the first neck cone to the base of the neck. Breast width **(BW)**: Measured with calipers as the distance between the 2 shoulder joints. Shank Length **(SL)**: Measured with calipers as the straight-line distance from the upper joint of the tibia to the midpoint between the third and fourth toes. Fossil bone length **(FBL)**: Measured with a tape measure as the distance between the 2 ends of the keel bone.

*Slaughter trait.* Slaughter Weight **(SW)**: The weight of the duck after 6 h of fasting before slaughter. Dressed Weight **(DW)**: The weight of the carcass after bloodletting and removal of feathers, foot cuticles, toe shells, and beak shells. Half-eviscerated weight **(H-EW)**: The weight of the carcass after removal of the trachea, esophagus, crop, intestines, spleen, pancreas, gallbladder, reproductive organs, gizzard contents, and keratin membrane. Eviscerated Weight **(EW**): Half-eviscerated weight minus heart, liver, proventriculus, gizzard, and abdominal fat. Abdominal fat weight **(AFW)**: Weight of abdominal fat and fat around the muscular stomach. Skin fat weight **(SFW)**: Weight of skin and subcutaneous fat. The dressed percentage **(DP)** was calculated as the DW / SW × 100%. The percentage of half-eviscerated yield **(H-EYP)** was calculated as the H-EW/SW × 100%. The eviscerated yield percentage (EYP) was calculated as the EW/SW × 100%. The breast muscle percentage (BMP) was calculated as the breast muscle weight **(BMW)**/EW × 100%. The leg muscle percentage **(LMP)** was calculated as the leg muscle weight **(LMW)**/EW × 100%. The abdominal fat percentage **(AFP)** was calculated as the AFW/(EW+AFW) × 100%. The skin fat percentage **(SFP)** was calculated as (SFW + AFW) / EW × 100%.

### DNA Extraction and Whole-Genome Re-Sequencing

The DNA samples were all extracted from duck whole blood using a standard Phenol-Chloroform extraction protocol. The quality of DNA was assessed using a NanoDrop2000 (Thermo. NanoDrop2000C) instrument and 0.8% agarose gel electrophoresis. After the examinations, standard procedures were used to generate paired-end libraries for each eligible sample. The average insert size was 500 bp, and the average read length was 150 bp. All libraries were sequenced on an Illumina HiSeq X-Ten platform or HiSeq 4000 platform to an average raw read sequence coverage of 5 × for the duck population. The raw reads were filtered using the NGS QC (v2.3.3) Toolkit with the default parameter. The depth ensured the accuracy of variant calling and genotyping and met the requirements for population genetic analyses.

### Genomic SNP calling

The raw sequence data was aligned to the duck reference genome (ZJU 1.0, RefSeq assembly accession: GCF_015476345.1) using Burrows-Wheeler alignment (BWA aln) with default parameters (Li and Durbin, 2009). SNP calling was performed exclusively using GATK (version 3.5) (DePristo, et al., 2011), and the output was further filtered using VCFtools (version 0.1.15) (Danecek, et al., 2011). The SNPs were filtered based on the following criteria: (1) the SNPs were required to have a minor allele frequency > 0.03 (2) the maximum missing rate was < 0.1; and (3) the SNPs with only 2 alleles; (4) SNPs with heterozygosity greater than 80% were removed. After filtering, 17,967,246 SNPs were finally obtained and used for the subsequent analysis.

### SNP-Based Heritability Calculation

Using genotypic and phenotypic data, a genetic relationship matrix (**GRM**) was constructed using Plink software. By combining GRM and phenotypic data, Genome-wide Complex Trait Analysis (**GCTA**) was used to partition the phenotypic variance into genetic and nongenetic parts to estimate heritability.

### GWAS

GWAS was performed on the Sichuan Shelduck population to detect genomic regions that affect growth and meat production traits in ducks using the mixed linear model program EMMAX (Kang, et al., 2010). The random effect was the phylogenetic matrix estimated by all genome-wide SNPs. The whole-genome significance cutoff was defined as the *Bonferroni* threshold, 0.05 / Total SNPs (−log_10_ (*P*) = 8.56). The linear model is as follows:

y=Xα+Zβ+Wμ+e

where *y* is the vector of phenotypic values of indicators, *Xα* is the fixed effects. To reduce the impact of gender on the results, “gender” was used as a covariate. *Zβ* represents the effect of SNP, and *β* represents the allele substitution effect. *Wμ* represents random animal effects with a variance–covariance structure based on the kinship matrix estimated using whole-genome SNP genotypes, and *e* is random residuals for data.

The Manhattan plots were generated using R (v3.5.1) and the ‘qqman’ package (Turner, 2018). The QQ plots were also generated to detect false positives from population stratification. The QQ plot shows that the ordinate represents the observed SNP *p*-value, while the abscissa represents the theoretical *p*-value generated using a chi-squared distribution. SNP variant information was combined to screen for potential molecular markers. Gene annotation was performed using SnpEff software, and individual genotypes for candidate regions were extracted using VCFtools in a Linux environment.

### Candidate Gene Selection

The KEGG and GO enrichment was conducted using KOBAS 3.0 online tools (https://bioinfo.org/kobas/gene list/). Finally, we performed GO and KEGG analysis and visualization using R software packages “cluster Profiler”, “enrich plot”, and “ggplot2”.

### Statistical Analysis

The study utilized statistical methods to calculate the mean and standard deviation for various phenotypic data. An inter-group difference analysis was conducted using independent sample *T*-tests with SPSS to assess differences in slaughter indicators among different genders in Sichuan Shelduck. Spearman was used to perform a correlation analysis between growth rate and meat production performance.

## RESULTS

### Descriptive Statistics

**Figure 1:**
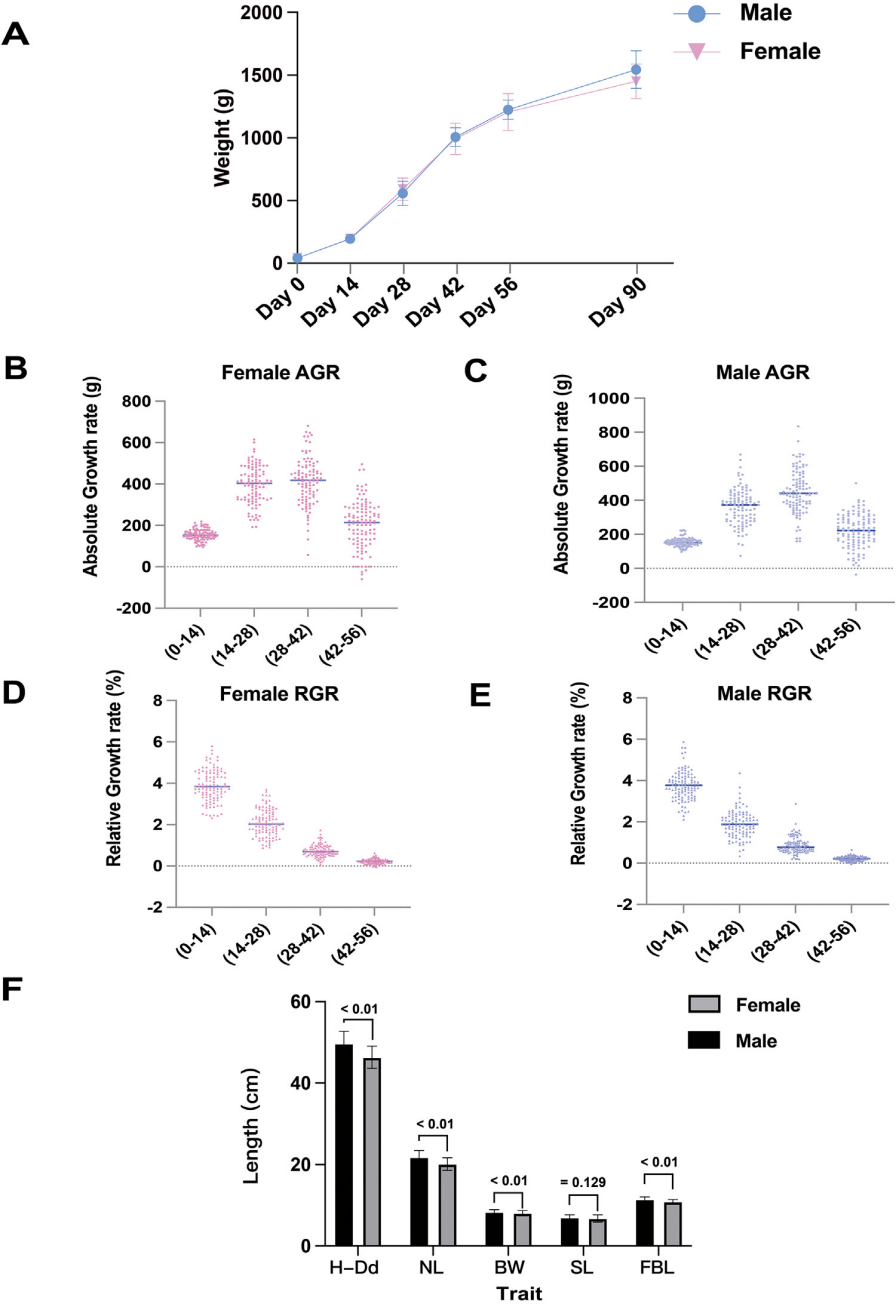
The descriptive statistics of phenotypic traits of Sichuan Shelducks. (A) Male and female ducks' body weight growth curve from birth to 90 d old. The X-axis and Y-axis showed the ages and body weights, respectively. (B–E) Absolute growth rate of female and male ducks for each growth stage, respectively. Dots in the bar graphs represent the RGR or AGR of the individual sex group. x- and y-axes represent different growth stages and growth rate data. (F) Comparison of body size differences between males and females of Sichuan Shelduck. H-Dd, Half-Divina dept; NL, Neck length; BW, Breast width; SL, Shank length; FBL, Fossil bone length.

To better evaluate the growth rate of the research population, we collected the body weight data of ducks every 2 wk from hatching to 56 d of age (Supplementary Table S2). At 90 d of age, the body weight was 1556 ± 139.5 g for males and 1459 ± 136.29 g for females. The growth curve results indicate that ducks exhibit relatively rapid growth before 42 d, gradually slowing down after 56 d of age (Figure 1A, Supplementary Table S3). In addition, we calculated the RGR and AGR for all ducks at 4 stages before 56 d of age. The RGR and AGR patterns were slightly different during the peak growth period. The peak of AGR appeared at 28 to 42 d of age. Both male and female ducks showed decreasing growth for the first time at 42 to 56 d of age (Figures 1B and 1C). The RGRs of the population reached a maximum at the beginning and then decreased gradually (Figures 1D and 1E). The body size results of male and female ducks showed that all body size indicators showed significant differences between male and female ducks (*P* < 0.01), except SL (Figure 1F).

In this study, 16 slaughter performance indexes of Sichuan Shelducks were measured and calculated (Table 1). The results showed that the slaughter weight of male ducks was 1520.67 ± 152.95 g, and the female ones were 1429.74 g. The DP of male ducks was 78.029%, and the female ones were 79.005%. The DP and H-EYP reached more than 70%. The BMP and LMP were 10.84% ​​and 12.60%, respectively. The average LP of ducks was 23.43%. In addition, there were significant differences between males and females in some carcass indicators, including EW, LMW, AFW, BMP, SFP and LP (*P* < 0.05).

**Table 1:** Comparison of carcass phenotypes between male and female ducks.

Trait type	Trait	Males		Female		*P*
		N	Mean ± SD	N	Mean ± SD	
Carcass yield	SW(g)	120	1540.92 ± 148.76	113	1451.42 ± 139.60	<0.01
	DW(g)	120	1202.83 ± 131.39	113	1147.52 ± 129.74	<0.01
	DP (%)	120	78.03 ± 0.03	113	79.01 ± 0.04	<0.05
	H-EW (g)	119	1104.71 ± 123.34	113	1053.01 ± 126.01	<0.01
	H-EYP (%)	119	71.61 ± 0.03	113	72.48 ± 0.04	0.05
	EW (g)	118	1004.24 ± 114.22	113	960.35 ± 111.73	<0.01
	EYP (%)	118	65.10 ± 0.03	113	66.12 ± 0.04	<0.05
	LMW (g)	120	126.135 ± 15.64	113	120.39 ± 15.70	<0.01
	LMP (%)	118	12.62 ± 0.01	113	12.59 ± 0.01	0.9
	BMW (g)	120	104.88 ± 22.02	113	109.52 ± 19.06	0.09
	BMP (%)	118	10.40 ± 0.01	113	11.38 ± 0.01	<0.01
	AFW (g)	86	6.28 ± 5.01	96	8.91 ± 5.62	<0.01
	AFP (%)	85	0.58 ± 0.00	96	0.88 ± 0.00	<0.01
	SFW (g)	119	187.32 ± 177.84	113	179.61 ± 43.98	0.65
	SFP (%)	85	20.65 ± 0.22	96	19.95 ± 0.04	0.76
	LP (%)	118	23.01 ± 0.02	113	23.97 ± 0.02	<0.01
Internal organs (g)	GW	120	46.91 ± 7.64	113	39.12 ± 6.32	<0.01
	GSW	119	4.28 ± 0.78	113	4.08 ± 0.91	0.08
	HW	120	9.83 ± 1.49	113	9.41 ± 1.19	<0.05
	LW	120	34.33 ± 4.99	113	32.14 ± 5.01	<0.01
	SW	115	0.58 ± 0.24	106	0.58 ± 0.26	0.89

Note: SW, Slaughter weight; DW, Dressed weight; DP, Dressed percentage; H-EW, Half-eviscerated weight; H-EYP, Percentage of half-eviscerated yield; EW, Eviscerated weight; EYP, Percentage of eviscerated yield; LMW, Leg muscle weight; LMP, Leg muscle percentage; BMW, Breast muscle weigh; BMP, Breast muscle percentage; AFW, Abdominal fat weight; AFP, Abdominal fat percentage; SFW, Skin fat weight; SFP, Skin fat percentage; LP, Percentage of lean meat BMT, Breast muscle thickness; GW, Gizzard weight; GSW, Glandular stomach weight; HW, Heart weight; LW, Liver weight; SW, Spleen weight. N is the number of samples; the mean is the average value; S.D. is the standard deviation. The *P-*values by *T*-test for the significance between groups of males and females were provided.

### Phenotypic Correlation Analysis

**Figure 2:**
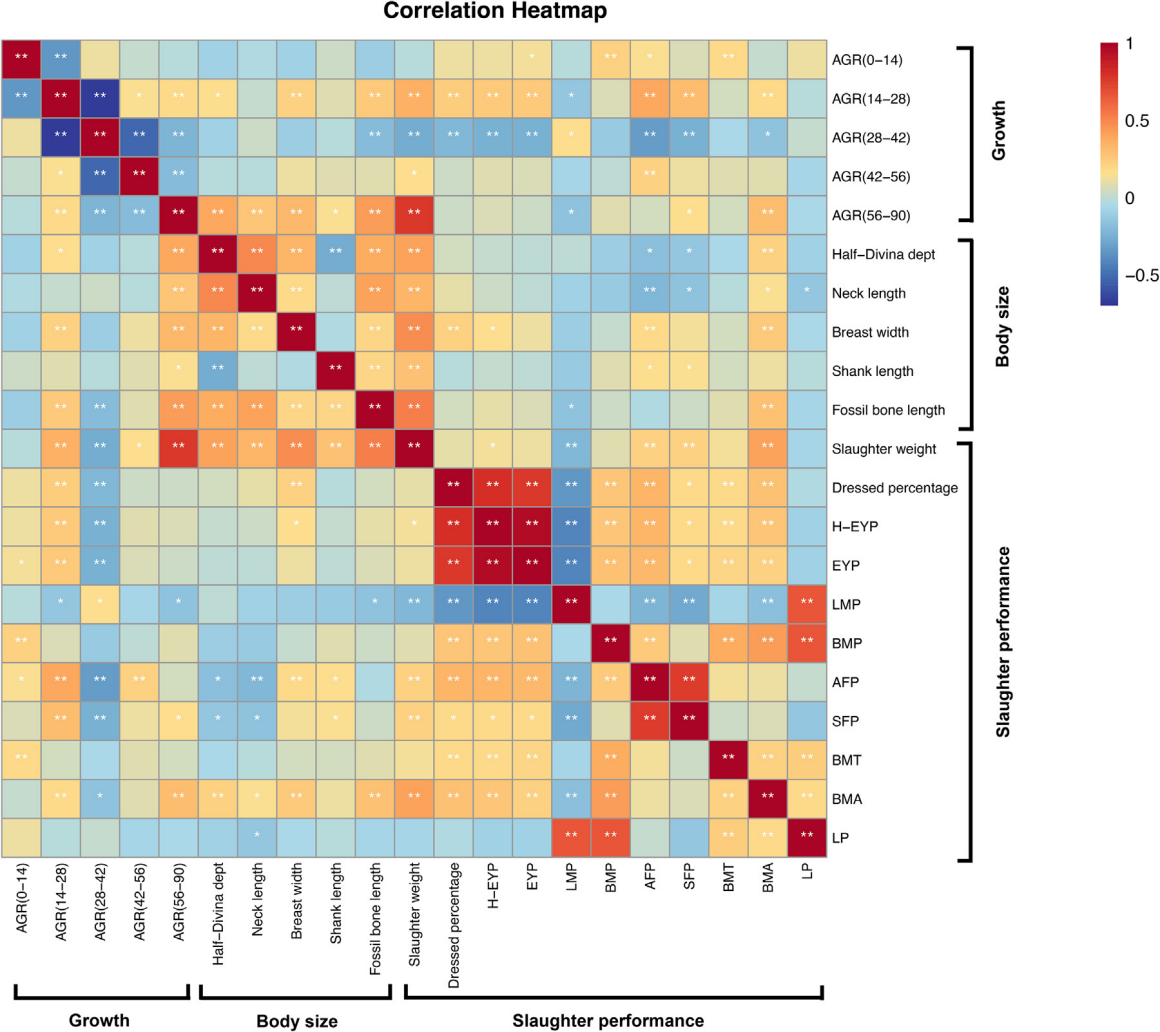
Correlation analysis chart among phenotypic indicators. H-EYP, percentage of half-eviscerated yield; EYP, Eviscerated yield percentage; LMP, Leg muscle percentage; BMP, Breast muscle percentage; AFP, Abdominal fat percentage; SFP, Skin fat percentage; LP, Percentage of lean meat; BMT, Breast muscle thickness; BMA, Breast muscle area. The depth of colors in the chart indicates the strength of the correlation, with positive and negative correlations represented by warm and cool colors, respectively. Asterisks serve as markers for significance, with more asterisks indicating higher significance. Deeper colors and more asterisks signify a stronger correlation. A single table is diagonally symmetrical, with diagonal values set to 1. ** *P* < 0.01 and * *P* < 0.05.

The results of the phenotypic correlation analysis showed a significant correlation between meat production performance and body size development indicators (Figure 2, Supplementary Table S4). BW showed substantial correlations with DP (R = 0.218, *P* < 0.01) and H-EYP (R = 0.166, *P* < 0.05). In terms of growth and development, AGR (14–28), AGR (28–42), and AGR (56–90) were significantly correlated with the FBL in body size (*P* < 0.01). This suggests that FBL may be an important factor influencing growth rates during these periods. The AGR (28–42) was negatively correlated with most traits, except for a positive correlation with LMP. Additionally, LMP showed a significant negative correlation with AGR (14–28). It can be inferred that the growth in weight from 28 to 42 d of age primarily depends on the development of leg muscles. The correlation analyses further revealed significant correlations between different meat production phenotypes such as AFP and H-EYP (R = 0.344), EYP (R = 0.327) and LMP (R = −0.222) (*P* < 0.01).

### Genetic Parameter Estimation Based on SNPs

**Figure 3:**
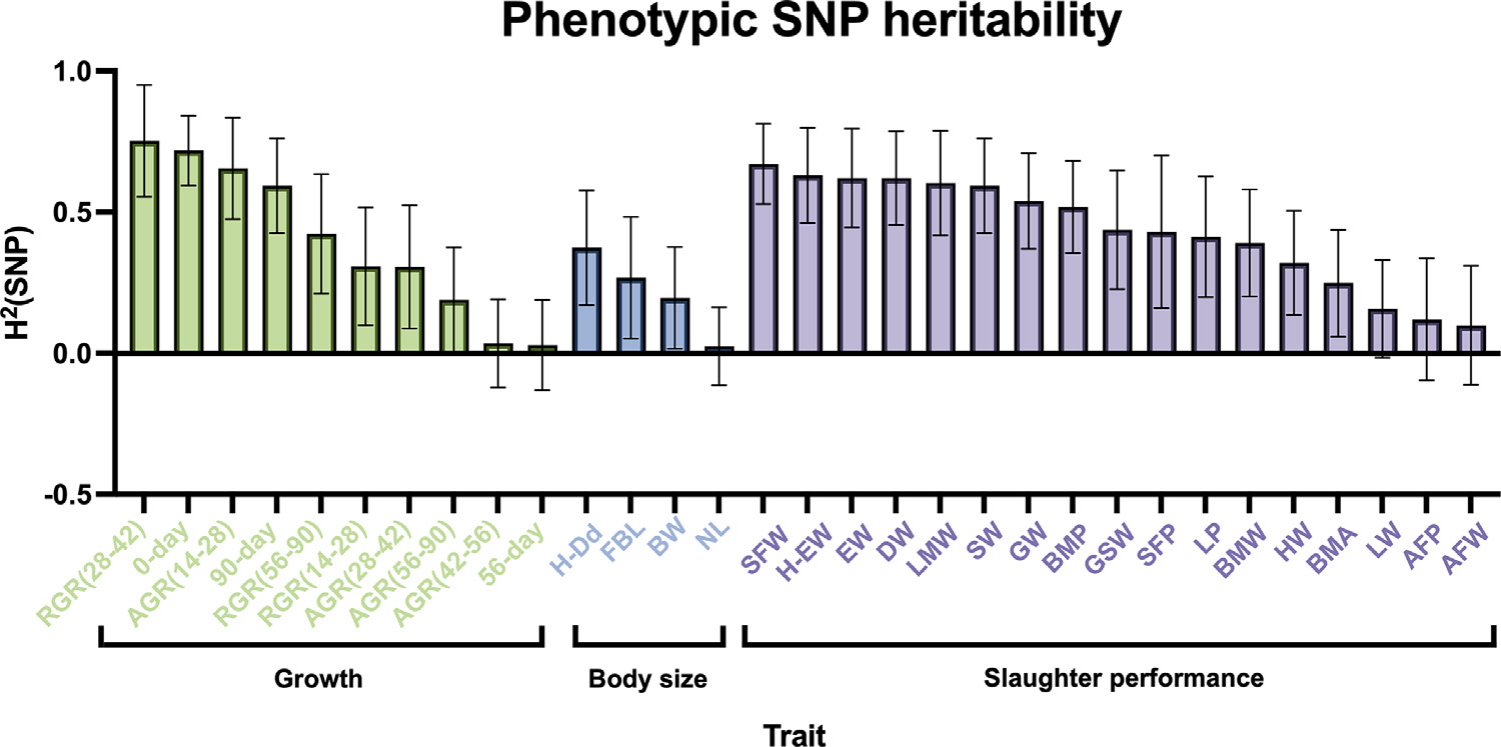
SNP-based genetic parameter estimation The Y-axis represents SNP heritability, and the X-axis represents phenotype. The higher the bar, the higher the heritability.

The heritability based on SNP is the extent to which it affects an individual trait or a specific genetic characteristic, reflecting the strength of association between the genotype and phenotype of the SNP. The SNP-based heritability estimation showed (Figure 3, Supplementary Table S5) that the heritability differs among stages. Among the growth traits, RGR (28–42) has the highest heritability, followed by 0-day-old weight, AGR (14–28), and 90-day-old, all exceeding a heritability of 0.5. Body size traits with higher heritability included H-Dd (0.37) and FBL (0.27). Regarding slaughter traits, the heritability for 8 indicators, SFW, H-EW, EW, DW, LMW, SW, GW, and BMP exceeded 0.5. This may indicate that genetic factors largely influence these traits.

### GWAS Screening for Candidate Genes Related to Production Performance

The GWAS results were derived from a dataset comprising 17,967,246 genomic SNP. We conducted GWAS analysis on 6 growth development and slaughter performance phenotypes: AGR (42–56), AGR (56–90), EW, BMW, AFW, and LP. The top 20 SNP in the GWAS results were selected for gene annotation.

### Growth

**Figure 4:**
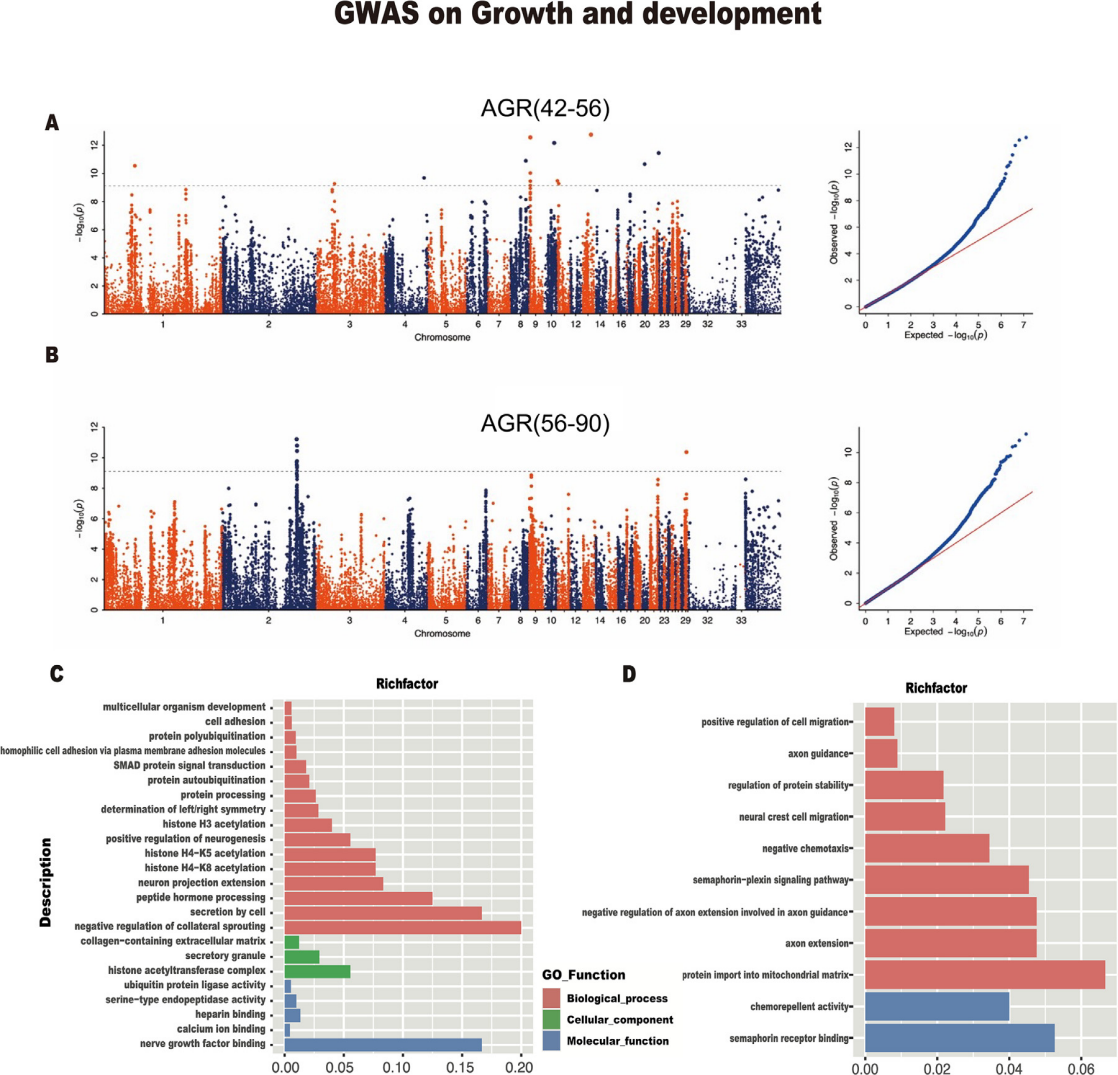
The Manhattan and quantile**-**quantile (QQ) plots of genome-wide association analysis of AGR (42-56), AGR (56-90), and GO functional enrichment analysis plots. A Manhattan and QQ plots of AGR (42-56). B, Manhattan and QQ plots of AGR (56-90). Each point represents one SNP, and abscissa numbers represent different chromosomes. The dotted line in the Manhattan plot represents the threshold level. C, GO enrichment of genes identified in AGR (42-56). D, GO enrichment of genes identified in AGR (56-90). Each row represents an enriched function, and the length of the bar represents the enrich ratio, which is calculated as “input gene number”/“background gene number”. The bar's color is the same as the color in the circular network above, representing different clusters.

Based on the GWAS analysis, the AGR (42-56) results showed clear signals on the chromosomes in the Manhattan map (Figure 4A), with 21 SNP sites surpassing the threshold line. Through SNP annotation, we discovered *PCSK6, LOC101795342, TOX2, LOC101795089, JADE2, PCDH11X, LOC110352402, TOMM22, LOC106019054* and *FSTL4* genes (Supplementary Table S6). The AGR (56–90) GWAS analysis results showed 22 significant SNP sites above the threshold line (Figure 4B). These loci were associated with *TOMM7, SEMA5A, CDH22, LOC113842992* and *LOC101795342* genes according to the SNP annotation (Supplementary Table S6).

In addition, we performed GO enrichment analysis (Supplementary Table S7) on other identified genes to explore potential candidate genes responsible for duck growth rate. The GO terms are divided into 3 categories: Molecular Function (MF), Cellular Component (**CC**), and Biological Process (**BP**). In the AGR (42–56) group, multiple potential growth-related pathways and clusters were identified, including negative regulation of collateral sprouting, the nerve growth factor binding, secretion by cell, nerve growth factor binding, the multicellular organism development, among others, in which the *PCSK6, JADE2,* and *PCDH11X* genes were enriched (Figure 4C). In the GO analysis results of AGR (56-90), *TOMM7* was found to be enriched in protein import into the mitochondrial matrix, regulation of protein stability, and integral component of membrane. The *SEMA5A* gene was also enriched in 9 other pathways (Figure 4D).

### Slaughter Performance

**Figure 5:**
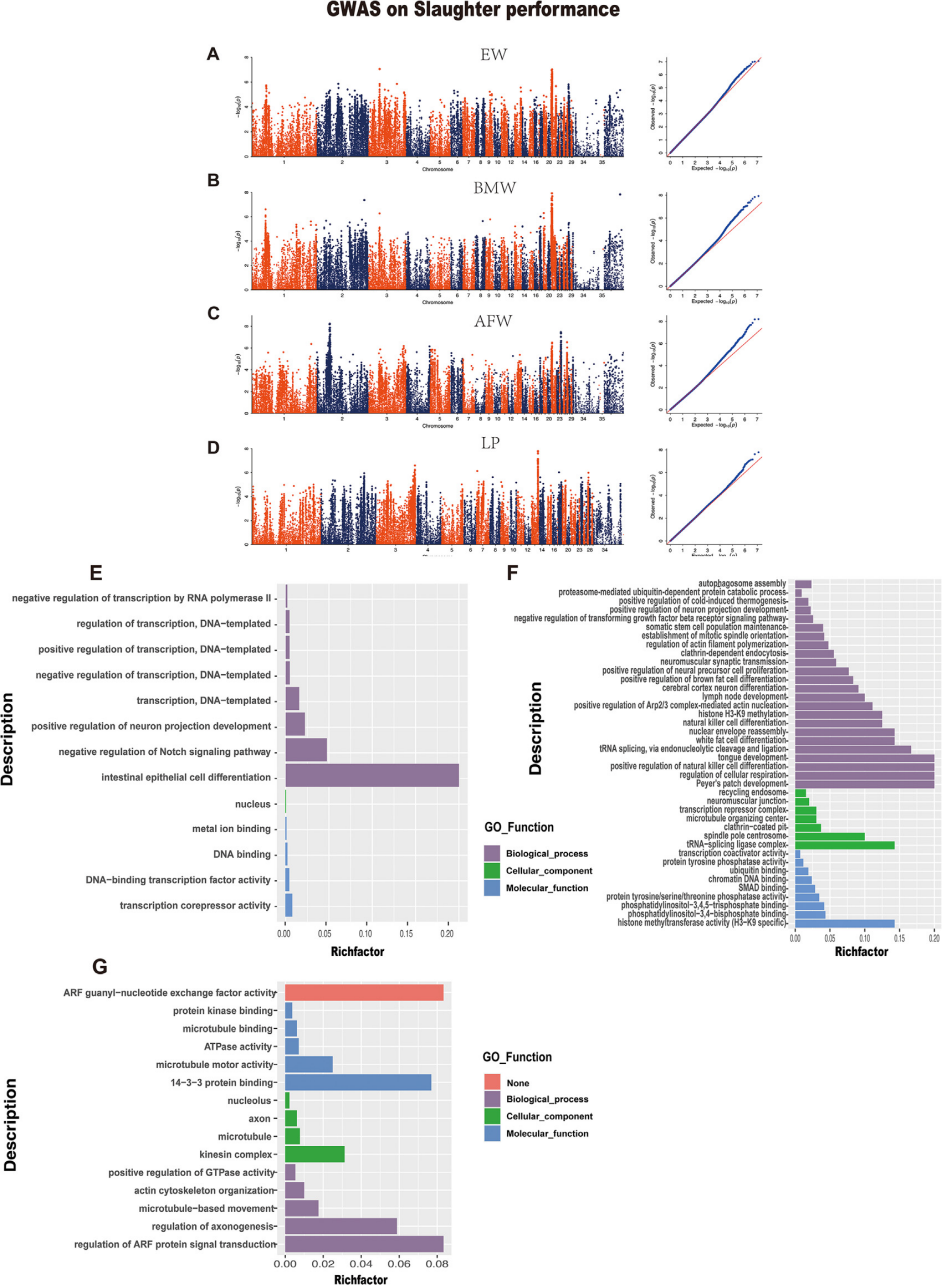
The Manhattan and quantile-quantile (QQ) plots of genome-wide association analysis of EW, BMW, AFW, LP, and GO functional enrichment analysis plots. A, B, C, and D, Manhattan plot of EW, BMW, AFW, and LP respectively, in slaughter performance indicators. Each point represents one SNP, and abscissa numbers represent different chromosomes. The dotted line in the Manhattan plot represents the threshold level. E, Plot of GO functional enrichment analysis of candidate genes in EW and BMW. F, Plot of GO functional enrichment analysis of candidate genes in AFW. G, Plot of GO functional enrichment analysis of candidate genes in LP. Each row represents an enriched function, and the length of the bar represents the enrich ratio, which is calculated as “input gene number”/“background gene number”. The bar's color is the same as the color in the circular network above, representing different clusters.

GWAS results showed potential signals for 4 traits (EW, BMW, AFW, LP); however, no significant results were found after the *Bonferroni* correction. Therefore, we selected the top 20 SNPs for each of the 4 traits for annotation to explore candidate genes affecting meat performance (Supplementary Table S8). Specifically, the GWAS analysis results of EW and BMW identified (Figures 5A, 5B) a total of 40 SNPs associated with these 2 phenotypes (Chr3: 36123219 C>G, chr21: 3347530 T>C, chr21: 2334214 G>T, etc.). These SNPs were annotated to 5 genes, including *LOC110352184, ASIP, ZNF341, CBFA2T2,* and *LOC101802175. TMEM68, TOX, FAM110B, PTP4A2*, and *UBXN2B* genes were annotated in AFW, while the *IQSEC1* and *KIF13B* genes were annotated in the LP.

We then performed GO enrichment analysis on these genes to explore potential genes that determine duck meat production performance (Figures 5E–G, Supplementary Table S9). The findings indicated that the gene *CBFA2T2*, identified through annotation of SNPs in EW, exhibited enrichment in 3 significant GO terms: intestinal epithelial cell differentiation, negative regulation of the Notch signaling pathway, and positive regulation of neuron projection development. These terms fall within the domain of biological functions and are likely associated with animal development and nerve growth (Figure 5E). In abdominal fat, we identified multiple pathways related to fat deposition, including white fat cell differentiation and positive regulation of brown fat cell differentiation, among which the *PRDM16* gene was enriched. In addition, the *FCHSD2* gene was enriched in the positive regulation of Arp2/3 complex-mediated actin nucleation and regulation of actin filament polymerization pathways, which are related to actin. In addition, the gene *IQSEC1* annotated in the LP indicator was enriched in actin cytoskeleton organization.

## DISCUSSION

Growth and carcass traits are crucial components for the profitability of the duck meat production industry to meet the increasing need for top-notch yet cost-effective poultry meat. Understanding the genetic foundations of these traits can enhance meat yield and quality, ultimately boosting financial gains and ensuring long-term sustainability in the industry. Sichuan Shelduck has an important genetic resource, but its phenotypic and genetic characteristics are still unclear. In this study, we evaluated its phenotypic and genetic parameters. In general, this breed is relatively small in body size and has a slow growth rate, which is possibly helpful for balancing body weight and bone development (Jain and Singhal, 2012; Duggan, et al., 2016; Zhang, et al., 2019). Regarding heritability, animal breeding is a complex process, and pedigree errors are common in commercial breeding practices, which can lower the accuracy of genetic parameter estimation (Zhu, et al., 2019). Utilizing a relationship matrix based on high-density genotyping effectively addresses these errors (Goddard, et al., 2011; Munoz, et al., 2014). Our results showed that RGR (28–42) had the highest heritability, followed by day-old weight, AGR (14–28) (0.66), SFW (0.67). It indicates that these traits are genetically stable and predictable, and the selection potential for this trait could be high, implying significant improvement may be obtained during crossing with other varieties. Currently, there are limited studies on the heritability of slaughter performance indexes in Sichuan Shelducks. The present study is the first evaluation work on this breed, which helps to deepen the understanding of the genetic basis of growth and meat production in this breed. In addition, the results of the correlation analysis revealed that different tissue sites exhibit distinct developmental timelines. Notably, indicators such as breast muscle, abdominal fat and skin fat mainly develop early, while leg muscles develop later. We speculate that there may be temporal specificity in regulating gene expression in different muscle tissues. This result suggests that appropriate time point sampling must be considered during future studies of gene expression regulation in the breast and leg muscles.

Animal growth is a complex physiological process influenced by various factors (Hu, et al., 2015). This study identified multiple SNPs associated with growth weight traits in 240 Sichuan Shelduck. Among all the identified SNPs, 41 were found in the GWAS results for AGR (42–56) and AGR (56–90), annotated to 6 genes, with *PCSK6* being the most significant. *PCSK6*, identified to be highly expressed in muscle tissue, encodes a protease involved in muscle cell differentiation, proliferation, and apoptosis, which is crucial for regulating muscle growth (Rykaczewska, et al., 2020). *PCSK6* gene knockout in mice results in abnormal bone development (Malfait, et al., 2012). Additionally, *PCSK6* may affect energy metabolism regulation (Li, et al., 2023a). LH-regulated *PCSK6* inhibits apoptosis of human granulosa cells through activin A and TGFb2 activation (Wang, et al., 2014). Overall, the impact of the *PCSK6* gene on animal growth is multifaceted. The *TOX2* gene primarily influences animal growth by promoting the differentiation of T follicular helper cells (Xu, et al., 2019), thus affecting the immune response through the proliferation and maturation of B lymphocytes. In the AGR (56–90) GWAS results, the *TOMM7* gene was found. *TOMM7*'s gene product, a subunit of the translocase of the outer mitochondrial membrane, regulates the assembly and stability of the translocase complex (Hönlinger, et al., 1996). *TOMM7* was identified to be associated with severe growth retardation and premature aging traits (Garg, et al., 2022). These genes may serve as key candidate genes in the later growth stages of Sichuan Shelducks.

In terms of meat production performance, the presence of the *CBFA2T2* gene was observed in the EW of Sichuan Shelduck. This gene is related to the weight of chickens at various growth stages (Gu, et al., 2011; Sharma, et al., 2015). The encoded product of *CBFA2T2* is believed to regulate skeletal development. *CBFA2T2* is essential for osteogenic differentiation of mesenchymal stem cells (Huang, et al., 2018). Moreover, *CBFA2T2* facilitates adipogenic differentiation of mesenchymal stem cells by regulating CEBPA (Luo, et al., 2020). It influences cell function and tissue development, including being a key regulator of fat generation (Luo, et al., 2020), suggesting an association with muscle growth, fat metabolism, and potential impact on meat production performance. Abdominal fat, the primary deposition site for body fat, is highly correlated with overall body fat. In the GWAS results of this study, AFW displayed a distinct signal, with annotated genes including *PTP4A2, FAM110B, TOX, UBXN2B, and FCHSD2. PTP4A2*, identified as a protein tyrosine phosphatase, demonstrates higher expression in female elephant adipose tissue than males (Nilsson, et al., 2014). It has been suggested that *PTP4A2/PRL2* may regulate leptin receptor signaling in adipose tissue (Huan, et al., 2003). *FAM110B* has been associated with the lean meat rate of pigs (Wang, et al., 2022). In addition, in animals (Machado, et al., 2022), *UBXN2B, TOX*, and *FAM110B* genes were related to carcass weight and quality (Alam, et al., 2023). For example, the *FAM110B* and *TOX* genes influence meat marbling (Li and Kim, 2015; Seabury, et al., 2017; Srikanth, et al., 2020). *TOX* and *FAM110B* impact reproductive traits in cattle(Fortes, et al., 2011), influencing maternal ability and calving ease in Nellore females (Silva, et al., 2020). *UBXN2B*'s enrichment in various cellular functional pathways underscores its importance in cell development (Machado, et al., 2022). *TOX, FAM110B,* and *UBXN2B* have been identified to affect multiple traits (Naserkheil, et al., 2022), including growth, birth weight, carcass weight, average daily gain, feed intake, meat tenderness, height, and stature, in different beef cattle breeds (Magalhães, et al., 2016; Seabury, et al., 2017; Brunes, et al., 2021). *FCHSD2*, involved in cytoskeletal remodeling (Abouelezz and Almeida-Souza, 2022), may play a role in processes such as cell proliferation, differentiation, and migration, which impact animal fat production and metabolism. Studies have linked *FCHSD2* to milk fat percentage (Marete, et al., 2018), although further research is needed for confirmation.

## CONCLUSION

In summary, Sichuan Shelducks are generally small in body size, and slow-growing rapid, with a body weight of 1497.91 g at 90 d of age. However, the performance of the meat was better. The DP and H-EYP reached more than 70%. The BMP and LMP were 10.84% ​​and 12.60%, respectively. The average LP of ducks was 23.43%. GWAS highlights *PCSK6, TOX2*, and *TOMM7* as candidate genes influencing later-stage growth (42–56, 56–90 d). *PTP4A2, FAM110B, TOX*, and *UBXN2B* also affect AFW, while *CBFA2T2* is associated with EW. The identified SNPs serve as valuable molecular markers for selecting growth rate and meat production traits, enhancing production efficiency for Sichuan Shelducks.

## DISCLOSURES

The authors declare no conflicts of interest.

## Appendix Supplementary materials

Image, application 1

Image, application 2

Image, application 3

Image, application 4

Image, application 5

Image, application 6

Image, application 7

Image, application 8

Image, application 9

## Footnotes

Supplementary material associated with this article can be found in the online version at doi:10.1016/j.psj.2024.104211.
