# Supplementary material for: Genome-wide association studies reveal the genetic basis of growth and carcass traits in Sichuan Shelduck
Source: Poult Sci. 2024 Aug 14;103(11):104211. doi: 10.1016/j.psj.2024.104211 (PMC11402601; doi:10.1016/j.psj.2024.104211)
Supplement: Supplementary file 1 [file mmc1.docx]

**Table S1 Duck feed composition**

| Feed main ingredients | Guaranteed values (%) |
| --- | --- |
| Crude protein | ≥20.0 |
| Crude fiber | ≤6.0 |
| Crude ash | ≤16.0 |
| Calcium | 2.70 - 4.00 |
| Total phosphorus | ≥0.60 |
| Sodium chloride | 0.20 - 0.80 |
| Methionine | ≥0.32 |
| Moisture | ≤12.0 |

Note: The guaranteed values in feed refer to the minimum or maximum content of various nutritional and other essential components listed on the label or product specification of feed products.
