# Supplementary material for: Genome-wide association studies reveal the genetic basis of growth and carcass traits in Sichuan Shelduck
Source: Poult Sci. 2024 Aug 14;103(11):104211. doi: 10.1016/j.psj.2024.104211 (PMC11402601; doi:10.1016/j.psj.2024.104211)
Supplement: Supplementary file 2 [file mmc2.docx]

**Table S2 Body weight of Sichuan Shelducks at different stages**

| **Age (days)** | **Male** | | **Female** | |
| --- | --- | --- | --- | --- |
|  | **N** | **Body weight (g)** | **N** | **Body weight (g)** |
| 0 (g) | 121 | 40.96±3.20 | 113 | 40.31±3.04 |
| 14 (g) | 111 | 194.15±23.96 | 107 | 195.44±26.84 |
| 28 (g) | 114 | 556.81±97.63 | 108 | 590.90±88.09 |
| 42 (g) | 136 | 1005.02±76.05 | 106 | 993.19±125.49 |
| 56 (g) | 137 | 1224.60±76.95 | 107 | 1205.71±148.49 |
| 90 (g) | 121 | 1543.47±150.16 | 113 | 1451.42±139.60 |
