# Supplementary material for: Genome-wide association studies reveal the genetic basis of growth and carcass traits in Sichuan Shelduck
Source: Poult Sci. 2024 Aug 14;103(11):104211. doi: 10.1016/j.psj.2024.104211 (PMC11402601; doi:10.1016/j.psj.2024.104211)
Supplement: Supplementary file 3 [file mmc3.docx]

**Table S3 Absolute growth rate and relative growth rate of Sichuan shelduck at various growth stages**

| **Growth rate (days)** | **Male** | | **Female** | |
| --- | --- | --- | --- | --- |
|  | **N** | **Mean ± SD** | **N** | **Mean ± SD** |
| AGR (0-14) | 110 | 153.52±24.05 | 107 | 155.24±26.97 |
| RGR (0-14) | 110 | 3.77±0.69 | 107 | 3.89±0.76 |
| AGR (14-28) | 103 | 362.51±104.88 | 102 | 395.42±92.37 |
| RGR (14-28) | 103 | 1.9±0.65 | 102 | 2.09±0.65 |
| AGR (28-42) | 111 | 430.13±177.03 | 102 | 400.91±157.56 |
| RGR (28-42) | 111 | 0.83±0.46 | 102 | 0.72±0.33 |
| AGR (42-56) | 118 | 224.67±134.43 | 108 | 221.71±145.69 |
| RGR (42-56) | 118 | 0.22±0.11 | 108 | 0.22±0.12 |

**Note:** AGR is the absolute growth rate; RGR is the relative growth rate.
