# Supplementary material for: Genome-wide association studies reveal the genetic basis of growth and carcass traits in Sichuan Shelduck
Source: Poult Sci. 2024 Aug 14;103(11):104211. doi: 10.1016/j.psj.2024.104211 (PMC11402601; doi:10.1016/j.psj.2024.104211)
Supplement: Supplementary file 5 [file mmc5.docx]

**Table S5. Table of SNP heritability for each phenotype.**

| Trait type | Trait | SNP heritability |
| --- | --- | --- |
| **Growth** | RGR (28-42) | 0.75±0.198 |
|  | 0-day old weight | 0.72±0.12 |
|  | AGR (14-28) | 0.66±0.180 |
|  | 90-day old weight | 0.59±0.17 |
|  | RGR (56-90) | 0.42±0.21 |
|  | RGR (14-28) | 0.31±0.21 |
|  | AGR (28-42) | 0.31±0.22 |
|  | AGR (56-90) | 0.19±0.19 |
|  | AGR (42-56) | 0.04±0.16 |
|  | 56-day old weight | 0.03±0.16 |
|  | AGR (0-14) | 0.000001 |
|  | RGR (42-56) | 0.000001 |
|  | RGR (0-14) | 0.000001 |
|  | 42-day old weight | 0.000001 |
|  | 14-day old weight | 0.000001 |
| **Body size** | H-Dd | 0.37±0.20 |
|  | FBL | 0.27±0.21 |
|  | BW | 0.20±0.18 |
|  | NL | 0.02±0.14 |
|  | SL | 0.000001 |
| **Slaughter performance** | SFW | 0.67±0.14 |
|  | H-EW | 0.63±0.17 |
|  | EW | 0.62±0.18 |
|  | DW | 0.62±0.17 |
|  | LMW | 0.60±0.19 |
|  | SW | 0.59±0.17 |
|  | GW | 0.54±0.17 |
|  | BMP | 0.52±0.16 |
|  | GSW | 0.44±0.21 |
|  | SFP | 0.43±0.27 |
|  | LP | 0.41±0.21 |
|  | BMW | 0.39±0.19 |
|  | HW | 0.32±0.18 |
|  | BMA | 0.24±0.19 |
|  | LW | 0.16±0.17 |
|  | AFP | 0.12±0.22 |
|  | AFW | 0.10±0.21 |
|  | DP | 0.000001 |
|  | H-EYP | 0.000001 |
|  | EYP | 0.000001 |
|  | LMP | 0.000001 |
|  | BMT | 0.000001 |
|  | SW | 0.000001 |

**Note:** SNP heritability, Mean±SE. The value 0.000001 indicates an infinitely small result from the calculation.
