# Supplementary material for: Genome-wide association studies reveal the genetic basis of growth and carcass traits in Sichuan Shelduck
Source: Poult Sci. 2024 Aug 14;103(11):104211. doi: 10.1016/j.psj.2024.104211 (PMC11402601; doi:10.1016/j.psj.2024.104211)
Supplement: Supplementary file 6 [file mmc6.docx]

**Table S6. The SNPs are significant for growth traits identified in GWAS.**

| **Trait** | **CHROME** | **POS** | **P** | **REF** | **ALT** | **Mutation type** | **Close protein coding gene** |
| --- | --- | --- | --- | --- | --- | --- | --- |
| **AGR (42-56)** | chr13 | 15694481 | 12.740 | T | C | intron_variant | PCSK6 |
|  | chr9 | 2700312 | 12.5451098 | A | T | upstream_gene_variant |  |
|  | chr10 | 17766516 | 12.1485494 | C | T | intron_variant | LOC101795342 |
|  | chr22 | 962644 | 11.4336174 | G | A | intron_variant | TOX2 |
|  | chr8 | 27278264 | 10.8851666 | G | C | 5_prime_UTR_variant | LOC101795089 |
|  | chr20 | 5214679 | 10.6479253 | G | A | upstream_gene_variant | JADE2 |
|  | chr1 | 53871574 | 10.5475743 | G | A |  |  |
|  | chr9 | 2691842 | 10.0150245 | C | T | downstream_gene_variant |  |
|  | chr4 | 69602415 | 9.66990369 | C | T | intergenic_region |  |
|  | chr11 | 915167 | 9.46486856 | C | T | intron_variant | PCDH11X |
|  | chr9 | 2689098 | 9.43877922 | G | T | upstream_gene_variant |  |
|  | chr11 | 3168894 | 9.26267314 | C | T | intergenic_region |  |
|  | chr3 | 32407893 | 9.26164723 | G | A | intron_variant | LOC110352402 |
|  | chr9 | 2691858 | 9.12974484 | C | T | upstream_gene_variant |  |
|  | chr9 | 2689125 | 8.92067197 | T | C | downstream_gene_variant |  |
|  | chr1 | 143503071 | 8.8470157 | T | C | upstream_gene_variant | TOMM22 |
|  | chr3 | 28412108 | 8.84289168 | G | A | intron_variant | LOC106019054 |
|  | chr14 | 2948800 | 8.77730475 | C | A | intron_variant | FSTL4 |
|  | chr3 | 28412125 | 8.69328053 | A | G | intron_variant | LOC110352402 |
|  | chr9 | 2690264 | 8.63954685 | G | A | downstream_gene_variant |  |
| **AGR (56-90)** | chr2 | 130857508 | 11.222 | T | C | upstream_gene_variant | TOMM7 |
|  | chr2 | 131503367 | 10.8017894 | C | T | downstream_gene_variant |  |
|  | chr2 | 131527831 | 10.4479249 | G | A | intergenic_region |  |
|  | chr29 | 3877592 | 10.3750659 | T | G | intergenic_region |  |
|  | chr2 | 131505139 | 9.78693922 | C | T | intergenic_region |  |
|  | chr2 | 130850698 | 9.73042339 | T | C | intergenic_region | SEMA5A |
|  | chr2 | 130833383 | 9.73025992 | G | A | intron_variant | LOC113842992 |
|  | chr2 | 130853782 | 9.55822184 | G | A | upstream_gene_variant |  |
|  | chr2 | 131530940 | 9.48545917 | T | G | intergenic_region |  |
|  | chr2 | 131601828 | 9.46738023 | C | T | intergenic_region |  |
|  | chr2 | 131532659 | 9.39144988 | C | G | intergenic_region |  |
|  | chr2 | 131505065 | 9.36763923 | C | T | intergenic_region |  |
|  | chr2 | 131505071 | 9.36763923 | A | G | intergenic_region |  |
|  | chr2 | 131523279 | 9.1407541 | A | G | intergenic_region | TOMM7 |
|  | chr2 | 130852517 | 8.96768016 | T | C | upstream_gene_variant |  |
|  | chr2 | 131504013 | 8.87626616 | A | C | intergenic_region | LOC101795342 |
|  | chr9 | 4367548 | 8.86125674 | G | T | intron_variant |  |
|  | chr9 | 4367544 | 8.85441753 | T | C | intergenic_region |  |
|  | chr2 | 131582321 | 8.76068744 | T | C | intergenic_region | LOC101795342 |
|  | chr9 | 4367560 | 8.73225051 | T | C | intron_variant |  |
|  | chr2 | 131582265 | 8.58292183 | G | C | intergenic_region |  |
|  | chr21 | 16712474 | 8.57035213 | G | C | intron_variant | CDH22 |

**Note**: **CHROM**, the chromosome; **POS**, the position of SNP on chromosome; **Ref**, the base of reference genome; **Alt**, the mutated base.
