# Supplementary material for: Genome-wide association studies reveal the genetic basis of growth and carcass traits in Sichuan Shelduck
Source: Poult Sci. 2024 Aug 14;103(11):104211. doi: 10.1016/j.psj.2024.104211 (PMC11402601; doi:10.1016/j.psj.2024.104211)
Supplement: Supplementary file 7 [file mmc7.docx]

**Table S7. GO functional enrichment analysis for growth traits.**

| **Trait** | **ID** | **Gene Number** | **Background number** | **Rich factor** | **P-Value** | **Input** | **GO_Function** |
| --- | --- | --- | --- | --- | --- | --- | --- |
| **AGR (42-56)** | GO:0048671 | 1 | 5 | 0.2 | 0.001258284 | FSTL4 | Biological_process |
|  | GO:0032940 | 1 | 6 | 0.166666667 | 0.001467874 | PCSK6 | Biological_process |
|  | GO:0048406 | 1 | 6 | 0.166666667 | 0.001467874 | PCSK6 | Molecular_function |
|  | GO:0016486 | 1 | 8 | 0.125 | 0.00188695 | PCSK6 | Biological_process |
|  | GO:1990138 | 1 | 12 | 0.083333333 | 0.00272468 | JADE2 | Biological_process |
|  | GO:0043982 | 1 | 13 | 0.076923077 | 0.002934024 | JADE2 | Biological_process |
|  | GO:0043981 | 1 | 13 | 0.076923077 | 0.002934024 | JADE2 | Biological_process |
|  | GO:0005509 | 2 | 447 | 0.004474273 | 0.003410764 | PCDH11X\|FSTL4 | Molecular_function |
|  | GO:0050769 | 1 | 18 | 0.055555556 | 0.00398022 | JADE2 | Biological_process |
|  | GO:0000123 | 1 | 18 | 0.055555556 | 0.00398022 | JADE2 | Cellular_component |
|  | GO:0043966 | 1 | 25 | 0.04 | 0.005443417 | JADE2 | Biological_process |
|  | GO:0030141 | 1 | 34 | 0.029411765 | 0.007322144 | FSTL4 | Cellular_component |
|  | GO:0007368 | 1 | 35 | 0.028571429 | 0.007530716 | PCSK6 | Biological_process |
|  | GO:0016485 | 1 | 38 | 0.026315789 | 0.008156222 | PCSK6 | Biological_process |
|  | GO:0051865 | 1 | 48 | 0.020833333 | 0.010238964 | JADE2 | Biological_process |
|  | GO:0060395 | 1 | 55 | 0.018181818 | 0.011694801 | JADE2 | Biological_process |
|  | GO:0008201 | 1 | 75 | 0.013333333 | 0.015844897 | PCSK6 | Molecular_function |
|  | GO:0062023 | 1 | 82 | 0.012195122 | 0.017294132 | PCSK6 | Cellular_component |
|  | GO:0007156 | 1 | 100 | 0.01 | 0.021012898 | PCDH11X | Biological_process |
|  | GO:0004252 | 1 | 101 | 0.00990099 | 0.021219166 | PCSK6 | Molecular_function |
|  | GO:0000209 | 1 | 106 | 0.009433962 | 0.022249982 | JADE2 | Biological_process |
|  | GO:0007155 | 1 | 166 | 0.006024096 | 0.034552179 | PCDH11X | Biological_process |
|  | GO:0007275 | 1 | 172 | 0.005813953 | 0.035775555 | FSTL4 | Biological_process |
|  | GO:0061630 | 1 | 187 | 0.0053 | 0.0388 | JADE2 | Molecular_function |
| **AGR (56-90)** | GO:0030150 | 1 | 15 | 0.066666667 | 0.001342479 | TOMM7 | Biological_process |
|  | GO:0030215 | 1 | 19 | 0.052631579 | 0.001677958 | SEMA5A | Molecular_function |
|  | GO:0048675 | 1 | 21 | 0.047619048 | 0.001845676 | SEMA5A | Biological_process |
|  | GO:0048843 | 1 | 21 | 0.047619048 | 0.001845676 | SEMA5A | Biological_process |
|  | GO:0071526 | 1 | 22 | 0.045454545 | 0.00192953 | SEMA5A | Biological_process |
|  | GO:0045499 | 1 | 25 | 0.04 | 0.00218107 | SEMA5A | Molecular_function |
|  | GO:0050919 | 1 | 29 | 0.034482759 | 0.002516408 | SEMA5A | Biological_process |
|  | GO:0001755 | 1 | 45 | 0.022222222 | 0.003857196 | SEMA5A | Biological_process |
|  | GO:0031647 | 1 | 46 | 0.02173913 | 0.003940965 | TOMM7 | Biological_process |
|  | GO:0007411 | 1 | 112 | 0.008928571 | 0.009461952 | SEMA5A | Biological_process |
|  | GO:0030335 | 1 | 124 | 0.008064516 | 0.01046412 | SEMA5A | Biological_process |
