# Supplementary material for: Genome-wide association studies reveal the genetic basis of growth and carcass traits in Sichuan Shelduck
Source: Poult Sci. 2024 Aug 14;103(11):104211. doi: 10.1016/j.psj.2024.104211 (PMC11402601; doi:10.1016/j.psj.2024.104211)
Supplement: Supplementary file 8 [file mmc8.docx]

**Table S8. The top 20 SNPs identified in the GWAS for Slaughter performance.**

| **Trait** | **CHROME** | **POS** | **P** | **REF** | **ALT** | **Mutation type Close protein coding gene** | |
| --- | --- | --- | --- | --- | --- | --- | --- |
| **EW** | chr3 | 36123219 | 7.05419977 | C | G | intron_variant | LOC110352184 |
|  | chr21 | 3347530 | 7.01346199 | T | C | intergenic_region | / |
|  | chr21 | 2334214 | 6.97367995 | G | T | 3_prime_UTR_variant | ASIP |
|  | chr21 | 3464509 | 6.81876547 | T | C | intron_variant | ZNF341 |
|  | chr21 | 3570126 | 6.69460735 | G | A | upstream_gene_variant | CBFA2T2 |
|  | chr21 | 3349664 | 6.65885352 | A | T | intergenic_region | / |
|  | chr21 | 2341989 | 6.61626197 | T | C | upstream_gene_variant | ASIP |
|  | chr21 | 3571185 | 6.60581439 | A | G | upstream_gene_variant | CBFA2T2 |
|  | chr21 | 3388532 | 6.52437436 | A | G | upstream_gene_variant | LOC101802175 |
|  | chr21 | 3570312 | 6.45395938 | T | C | upstream_gene_variant | CBFA2T2 |
|  | chr21 | 3570328 | 6.45395938 | G | A | upstream_gene_variant | CBFA2T2 |
|  | chr21 | 3570929 | 6.45130169 | T | C | upstream_gene_variant | CBFA2T2 |
|  | chr21 | 2382055 | 6.432159 | G | A | intergenic_region | / |
|  | chr21 | 3570216 | 6.37509702 | C | G | upstream_gene_variant | CBFA2T2 |
|  | chr21 | 3570370 | 6.3298 | T | C | upstream_gene_variant | CBFA2T2 |
|  | chr21 | 3570270 | 6.28521612 | A | G | upstream_gene_variant | CBFA2T2 |
|  | chr21 | 3566781 | 6.26697027 | C | T | intron_variant | CBFA2T2 |
|  | chr21 | 3388545 | 6.25660828 | T | C | upstream_gene_variant | LOC101802175 |
|  | chr21 | 3389708 | 6.21828621 | T | C | missense_variant | LOC101802175 |
|  | chr21 | 3586375 | 6.20144194 | G | A | intergenic_region | / |
| **BMW** | chr21 | 2974051 | 7.93526286 | T | C | intergenic_region | / |
|  | chr34 | 50774415 | 7.83711094 | / | / | #N/A | / |
|  | chr21 | 4115687 | 7.68141089 | T | C | intergenic_region | LOC110351346-exon-XR_005260461.1-1 |
|  | chr21 | 2370377 | 7.51763446 | C | G | intragenic_variant | LOC110352121 |
|  | chr21 | 2336654 | 7.36783711 | T | C | intron_variant | ASIP |
|  | chr2 | 152197965 | 7.36476236 | C | T | intergenic_region | / |
|  | chr21 | 3388532 | 7.34249397 | A | G | upstream_gene_variant | LOC101802175 |
|  | chr21 | 2334214 | 7.07130568 | G | T | 3_prime_UTR_variant | ASIP |
|  | chr21 | 2333453 | 7.06177572 | T | C | 3_prime_UTR_variant | ASIP |
|  | chr21 | 2333454 | 7.06177572 | G | C | 3_prime_UTR_variant | ASIP |
|  | chr21 | 3388545 | 7.03149355 | T | C | upstream_gene_variant | LOC101802175 |
|  | chr21 | 2332101 | 7.00739875 | A | G | upstream_gene_variant | ASIP |
|  | chr21 | 3347530 | 6.98148386 | T | C | intergenic_region | / |
|  | chr21 | 3587701 | 6.97104883 | G | A | intergenic_region | / |
|  | chr21 | 2336857 | 6.9666265 | A | G | intron_variant | ASIP |
|  | chr21 | 2333082 | 6.9289404 | G | A | 3_prime_UTR_variant | ASIP |
|  | chr21 | 2392727 | 6.76124795 | G | T | intergenic_region | / |
|  | chr21 | 2345389 | 6.75458305 | T | C | upstream_gene_variant | ASIP |
|  | chr21 | 2332649 | 6.75058476 | C | G | upstream_gene_variant | ASIP |
|  | chr21 | 2336884 | 6.74073582 | G | A | intron_variant | ASIP |
| **AFW** | chr2 | 41796379 | 8.22216492 | A | T | intron_variant | FAM110B |
|  | chr2 | 41285205 | 8.19889171 | A | G | intron_variant | TOX |
|  | chr2 | 42091302 | 7.87282671 | A | G | intergenic_region | / |
|  | chr2 | 41280201 | 7.69026551 | T | C | upstream_gene_variant | FCHSD2 |
|  | chr2 | 41778426 | 7.6522936 | G | A | intron_variant | FAM110B |
|  | chr2 | 41778448 | 7.54220249 | T | C | intron_variant | FAM110B |
|  | chr24 | 942683 | 7.45852521 | T | C | downstream_gene_variant | PRDM16 |
|  | chr24 | 1141475 | 7.34626015 | A | G | upstream_gene_variant | ZBTB8OS |
|  | chr2 | 42960775 | 7.26298605 | A | C | intergenic_region | / |
|  | chr2 | 42065793 | 7.09375681 | A | T | intron_variant | FAM110B |
|  | chr24 | 966376 | 7.03189812 | T | C | intergenic_region | / |
|  | chr2 | 41285248 | 7.02931398 | T | C | intron_variant | TOX |
|  | chr2 | 41319853 | 6.87762002 | C | T | intron_variant | TOX |
|  | chr2 | 41660496 | 6.83788779 | C | T | intron_variant | UBXN2B |
|  | chr2 | 41711860 | 6.83445919 | T | G | intragenic_variant | LOC119715715 |
|  | chr2 | 41796414 | 6.7434191 | T | G | intron_variant | FAM110B |
|  | chr2 | 42091318 | 6.70189074 | A | C | intergenic_region | / |
|  | chr24 | 1653994 | 6.66051555 | T | C | intron_variant | PTP4A2 |
|  | chr2 | 41604632 | 6.64036002 | G | A | intron_variant | TOX |
|  | chr2 | 41284746 | 6.61296152 | C | T | intron_variant | TOX |
| **LP** | chr13 | 19786854 | 7.78553594 | C | A | intergenic_region | / |
|  | chr13 | 19786847 | 7.61200631 | A | G | intergenic_region | / |
|  | chr13 | 19802265 | 7.14119341 | C | A | intergenic_region | / |
|  | chr13 | 19787775 | 7.11448695 | T | C | intergenic_region | / |
|  | chr13 | 19797947 | 7.06528085 | T | G | intergenic_region | / |
|  | chr13 | 19284967 | 7.00006259 | C | T | intron_variant | IQSEC1 |
|  | chr13 | 19787758 | 6.9420169 | A | G | intergenic_region | / |
|  | chr13 | 19786206 | 6.87630229 | G | A | intergenic_region | / |
|  | chr13 | 19798990 | 6.81730236 | T | C | intergenic_region | / |
|  | chr13 | 19798984 | 6.76558968 | T | C | intergenic_region | / |
|  | chr13 | 19783123 | 6.73817715 | A | C | intergenic_region | / |
|  | chr13 | 19771298 | 6.71559735 | T | G | intergenic_region | / |
|  | chr3 | 117434015 | 6.60286515 | T | G | intron_variant | KIF13B |
|  | chr13 | 19806244 | 6.51949313 | C | T | intergenic_region | / |
|  | chr13 | 19809620 | 6.48507015 | C | T | intergenic_region | / |
|  | chr13 | 19776822 | 6.43802843 | G | A | intergenic_region | / |
|  | chr13 | 19791846 | 6.28794576 | T | C | intergenic_region | / |
|  | chr3 | 117434025 | 6.23769348 | A | G | intron_variant | KIF13B |
|  | chr13 | 19804336 | 6.1908286 | A | G | intergenic_region | / |
|  | chr13 | 19792583 | 6.18300859 | G | A | intergenic_region | / |
