# Supplementary material for: Genome-wide association studies reveal the genetic basis of growth and carcass traits in Sichuan Shelduck
Source: Poult Sci. 2024 Aug 14;103(11):104211. doi: 10.1016/j.psj.2024.104211 (PMC11402601; doi:10.1016/j.psj.2024.104211)
Supplement: Supplementary file 9 [file mmc9.docx]

**Table S9. GO functional enrichment analysis for Slaughter performance.**

| **Trait** | **ID** | **Gene Number** | **Background number** | **Rich factor** | **P-Value** | **Input** | **GO_Function** |
| --- | --- | --- | --- | --- | --- | --- | --- |
| **EW and BMW** | GO:0003714 | 1 | 124 | 0.0081 | 0.0157 | CBFA2T2 | Molecular_function |
|  | GO:0003700 | 1 | 216 | 0.0046 | 0.0271 | ZNF341 | Molecular_function |
|  | GO:0003677 | 1 | 403 | 0.0025 | 0.0500 | ZNF341 | Molecular_function |
|  | GO:0046872 | 1 | 714 | 0.0014 | 0.0873 | CBFA2T2 | Molecular_function |
|  | GO:0005634 | 2 | 2478 | 0.0008 | 0.0302 | ZNF341\|CBFA2T2 | Cellular_component |
|  | GO:0060575 | 1 | 5 | 0.2000 | 0.0008 | CBFA2T2 | Biological process |
|  | GO:0045746 | 1 | 21 | 0.0476 | 0.0028 | CBFA2T2 | Biological process |
|  | GO:0010976 | 1 | 45 | 0.0222 | 0.0058 | CBFA2T2 | Biological process |
|  | GO:0006351 | 1 | 64 | 0.0156 | 0.0082 | CBFA2T2 | Biological process |
|  | GO:0045892 | 1 | 209 | 0.0048 | 0.0262 | CBFA2T2 | Biological process |
|  | GO:0045893 | 1 | 222 | 0.0045 | 0.0278 | ZNF341 | Biological process |
|  | GO:0006355 | 1 | 226 | 0.0044 | 0.0283 | ZNF341 | Biological process |
|  | GO:0000122 | 1 | 520 | 0.0019 | 0.0642 | CBFA2T2 | Biological process |
| **AFW** | GO:0046974 | 1 | 7 | 0.1429 | 0.0023 | PRDM16 | Molecular_function |
|  | GO:0043325 | 1 | 23 | 0.0435 | 0.0070 | FCHSD2 | Molecular_function |
|  | GO:0005547 | 1 | 24 | 0.0417 | 0.0073 | FCHSD2 | Molecular_function |
|  | GO:0008138 | 1 | 29 | 0.0345 | 0.0088 | PTP4A2 | Molecular_function |
|  | GO:0046332 | 1 | 35 | 0.0286 | 0.0105 | PRDM16 | Molecular_function |
|  | GO:0031490 | 1 | 42 | 0.0238 | 0.0126 | TOX | Molecular_function |
|  | GO:0043130 | 1 | 53 | 0.0189 | 0.0158 | UBXN2B | Molecular_function |
|  | GO:0004725 | 1 | 86 | 0.0116 | 0.0253 | PTP4A2 | Molecular_function |
|  | GO:0003713 | 1 | 140 | 0.0071 | 0.0407 | PRDM16 | Molecular_function |
|  | GO:0072669 | 1 | 7 | 0.1429 | 0.0023 | ZBTB8OS | Cellular_component |
|  | GO:0031616 | 1 | 10 | 0.1000 | 0.0032 | UBXN2B | Cellular_component |
|  | GO:0005905 | 1 | 27 | 0.0370 | 0.0082 | FCHSD2 | Cellular_component |
|  | GO:0005815 | 1 | 33 | 0.0303 | 0.0099 | UBXN2B | Cellular_component |
|  | GO:0017053 | 1 | 33 | 0.0303 | 0.0099 | PRDM16 | Cellular_component |
|  | GO:0031594 | 1 | 50 | 0.0200 | 0.0149 | FCHSD2 | Cellular_component |
|  | GO:0055037 | 1 | 65 | 0.0154 | 0.0192 | FCHSD2 | Cellular_component |
|  | GO:0048541 | 1 | 5 | 0.2000 | 0.0018 | TOX | Biological_process |
|  | GO:0043457 | 1 | 5 | 0.2000 | 0.0018 | PRDM16 | Biological_process |
|  | GO:0032825 | 1 | 5 | 0.2000 | 0.0018 | TOX | Biological_process |
|  | GO:0043586 | 1 | 5 | 0.2000 | 0.0018 | PRDM16 | Biological_process |
|  | GO:0006388 | 1 | 6 | 0.1667 | 0.0021 | ZBTB8OS | Biological_process |
|  | GO:0050872 | 1 | 7 | 0.1429 | 0.0023 | PRDM16 | Biological_process |
|  | GO:0031468 | 1 | 7 | 0.1429 | 0.0023 | UBXN2B | Biological_process |
|  | GO:0001779 | 1 | 8 | 0.1250 | 0.0026 | TOX | Biological_process |
|  | GO:0051567 | 1 | 8 | 0.1250 | 0.0026 | PRDM16 | Biological_process |
|  | GO:2000601 | 1 | 9 | 0.1111 | 0.0029 | FCHSD2 | Biological_process |
|  | GO:0048535 | 1 | 10 | 0.1000 | 0.0032 | TOX | Biological_process |
|  | GO:0021895 | 1 | 11 | 0.0909 | 0.0035 | TOX | Biological_process |
|  | GO:0090336 | 1 | 12 | 0.0833 | 0.0038 | PRDM16 | Biological_process |
|  | GO:2000179 | 1 | 13 | 0.0769 | 0.0041 | TOX | Biological_process |
|  | GO:0007274 | 1 | 17 | 0.0588 | 0.0053 | FCHSD2 | Biological_process |
|  | GO:0072583 | 1 | 18 | 0.0556 | 0.0056 | FCHSD2 | Biological_process |
|  | GO:0030833 | 1 | 21 | 0.0476 | 0.0064 | FCHSD2 | Biological_process |
|  | GO:0000132 | 1 | 24 | 0.0417 | 0.0073 | UBXN2B | Biological_process |
|  | GO:0035019 | 1 | 25 | 0.0400 | 0.0076 | PRDM16 | Biological_process |
|  | GO:0030512 | 1 | 39 | 0.0256 | 0.0117 | PRDM16 | Biological_process |
|  | GO:0010976 | 1 | 45 | 0.0222 | 0.0134 | TOX | Biological_process |
|  | GO:0120162 | 1 | 53 | 0.0189 | 0.0158 | PRDM16 | Biological_process |
|  | GO:0043161 | 1 | 108 | 0.0093 | 0.0316 | UBXN2B | Biological_process |
|  | GO:0000045 | 1 | 43 | 0.0233 | 0.0129 | UBXN2B | Biological_process |
| **LP** | GO:0032012 | 1 | 12 | 0.0833 | 0.0011 | IQSEC1 | Biological process |
|  | GO:0050770 | 1 | 17 | 0.0588 | 0.0015 | KIF13B | Biological process |
|  | GO:0007018 | 1 | 57 | 0.0175 | 0.0049 | KIF13B | Biological process |
|  | GO:0030036 | 1 | 100 | 0.0100 | 0.0085 | IQSEC1 | Biological process |
|  | GO:0043547 | 1 | 187 | 0.0053 | 0.0157 | IQSEC1 | Biological process |
|  | GO:0005871 | 1 | 32 | 0.0313 | 0.0028 | KIF13B | Cellular_component |
|  | GO:0005874 | 1 | 129 | 0.0078 | 0.0109 | KIF13B | Cellular_component |
|  | GO:0030424 | 1 | 163 | 0.0061 | 0.0137 | KIF13B | Cellular_component |
|  | GO:0005730 | 1 | 446 | 0.0022 | 0.0372 | IQSEC1 | Cellular_component |
|  | GO:0071889 | 1 | 13 | 0.0769 | 0.0012 | KIF13B | Molecular_function |
|  | GO:0003777 | 1 | 40 | 0.0250 | 0.0034 | KIF13B | Molecular_function |
|  | GO:0016887 | 1 | 140 | 0.0071 | 0.0118 | KIF13B | Molecular_function |
|  | GO:0008017 | 1 | 162 | 0.0062 | 0.0136 | KIF13B | Molecular_function |
|  | GO:0019901 | 1 | 270 | 0.0037 | 0.0226 | KIF13B | Molecular_function |
|  | GO:0005086 | 1 | 12 | 0.0833 | 0.0011 | IQSEC1 | / |
